# Reliability and Validity of the Japanese Version of the Barriers to Access to Care Evaluation Scale Version 3 for People With Mental Disorders: an Online Survey Study

**DOI:** 10.3389/fpsyg.2021.760184

**Published:** 2021-10-28

**Authors:** Minako Hongo, Fumiyo Oshima, Hirofumi Nishinaka, Mikuko Seto, Toshiyuki Ohtani, Eiji Shimizu

**Affiliations:** ^1^United Graduate School of Child Development, Osaka University, Kanazawa University, Hamamatsu University School of Medicine, Chiba University, and University of Fukui, Osaka, Japan; ^2^Research Center for Child Mental Development, Chiba University, Chiba, Japan; ^3^Center for Forensic Mental Health, Chiba University, Chiba, Japan; ^4^Safety and Health Organization, Chiba University, Chiba, Japan; ^5^Department of Cognitive Behavioral Physiology, Graduate School of Medicine, Chiba University, Chiba, Japan

**Keywords:** BACE v3, treatment stigma, service gap, mental health, validation study

## Abstract

It is a serious problem when people with mental disorders avoid, delay, discontinue, or do not use treatment and support, despite the existence of evidence-based treatment and support methods. In this study, we aimed to clarify the factor structure of BACE v3, a scale to measure barriers to accessing mental health care, and to examine its reliability and validity among Japanese people with mental disorders. An online survey with 268 participants, 20 years old and over, who had received care from mental health services in the past 12 months was conducted. Exploratory and confirmatory factor analysis (EFA and CFA) were used to examine the structure of the BACE v3. Internal consistency and test-retest reliability of all subscales were examined. Convergent validity [correlation of one of the subscales of the BACE v3, the treatment stigma subscale with the Stigma Scale for Receiving Psychological Help (SSRPH) and with the Internalized Stigma of Mental Illness Scale (ISMI)] was assessed. EFA identified two factors (treatment stigma and non-stigma), and the results suggested that the factor structure of the Japanese version of BACE v3 was similar to the original 2-factor structure. Regarding the CFA result, the goodness-of-fit indices showed marginal fit (root mean square error of approximation = 0.087; Tucker–Lewis index = 0.842; standardized root mean square residual = 0.078; comparative fit index = 0.86). The internal consistency of the treatment stigma subscale was α = 0.90, and the intraclass correlation coefficient was 0.76 (confidence interval: 0.70–0.81). The internal consistency of the non-stigma subscale was α = 0.83, and the intraclass correlation coefficient was 0.64 (confidence interval: 0.56–0.71). The score of the treatment stigma subscale was significantly and positively correlated with the SSRPH and ISMI. Thus, the BACE v3 has acceptable consistency, reliability and validity for the assessment of barriers to accessing mental health care including treatment stigma among people with mental disorders in Japan.

## Introduction

In terms of mental health, a “service gap,” defined as discrepancies in healthcare access and services and the use of such services, exists when, despite the existence of evidence-based treatment and support modalities, individuals with mental disorders avoid, delay, or discontinue treatment or support, or do not use them at all (Stefl and Prosperi, 1985). In a survey conducted in Japan between 2002 and 2006, for example, 83% of people who had experienced mental illness had never received any formal care, decreasing to 72% when the survey was repeated between 2013 and 2015 (Kawakami, 2016). In high-income nations, such as some European countries and the United States, 50.4–78.5% of individuals with mental disorders receive no treatment (Evans-Lacko et al., 2018), illustrating a global challenge presented by service gaps (Kohn et al., 2004). Several factors contribute to the avoidance of treatment and mental healthcare, in turn giving rise to service gaps.

A review of previous research identified 13 inhibiting factors among individuals with mental disorders, including stigma, confidentiality concerns, and the availability, accessibility, acceptability, and affordability of treatment options (Gulliver et al., 2010). Of these, stigma was the most prominent barrier to seeking mental health treatment (Gulliver et al., 2010; Clement et al., 2015). Stigma is defined as a negative understanding and perception of socially undesirable attributes (Goffman, 1963). Research has shown that there is often a great deal of stigma around mental disorders (Corrigan and Bink, 2016). Individuals with mental health problems not only deal with mental health symptoms, but also experience secondary effects of mental health stigma.

The stigma surrounding mental disorders takes two forms, depending on the focus of stigmatization, namely public stigma and self-stigma (Corrigan and Watson, 2002). Much of the research is about public stigma. Public stigma is held by the general public or society as a whole and includes stereotypes, prejudices, and discrimination directed toward a specific group of people, such as those who are mentally ill or people with disabilities, and the negative belief that such people are incompetent and weak (Corrigan and Watson, 2002). Self-stigma is held by mentally ill individuals toward themselves; it is the internalization of public stigma, depending on the situation, and the negative perception of oneself as an “incapable and embarrassing person” (Corrigan and Watson, 2002). For example, if individuals with mental disorders internalize public stigma and perceive that “patients with mental disorders are disdained and discriminated against,” they will develop self-stigma. The emotional response to stigma is shame, anger, worry, and depression and, as a result, stigma interferes with treatment, even though treatment is available, also known as treatment stigma. Treatment stigma is the perception that a person who seeks psychological treatment is socially undesirable (Vogel et al., 2006). It is important to understand the extent to which people with mental health problems feel stigmatized and to counsel them against stigma.

There are four scales that measure treatment stigma barriers, but three of them cannot be used with general mental health populations and are restricted to targeted populations and facilities (Kuhl et al., 1997; Britt et al., 2008; Pepin et al., 2009). One of the three scales targets adolescents; another targets military personnel undergoing treatment for PTSD and other conditions. The third scale is used in the context of psychotherapy (Kuhl et al., 1997; Britt et al., 2008; Pepin et al., 2009). The Barriers to Access to Care Evaluation scale version 3 (BACE v3) (Clement et al., 2012) is applicable to all mental health conditions and all types of mental health care. The BACE v3 was designed to measure the behavioral barriers by using specialized treatment facilities for mental healthcare. It comprises a subscale that measures treatment stigma, which impedes help-seeking behavior in patients with mental disorders, and another concerning both practical and attitudinal challenges that hinder help-seeking behaviors. The scale is easy to administer, with only 30 questions and comprehensive scoring, and could be used as a complete tool for evaluating resistance to seeking help. The BACE v3 has been standardized for India, Italy, Colombia, and China; however, there is no existing comprehensive scale for assessing treatment stigma in Japan. Consequently, this study aimed to investigate the factor structure of the Japanese version of the Barriers to Accessing Treatment Evaluation Scale (BACE v3), to confirm the internal consistency and reliability of each of the subscales and examine the validity of the treatment stigma subscale.

## Materials and Methods

### Sample and Recruitment

The research participants were male and female adults between the ages of 20 and 65 who had received treatment from mental health services or a department of psychiatry within the last 12 months or were presently undergoing treatment, and had access to the Internet, as the survey was to be completed online. Individuals with mental disorders are often stigmatized and can therefore be difficult to recruit in-person. However, online surveys can make it possible to communicate with people who may be hesitant to meet face-to-face (Wright, 2005), and to recruit participants based on illness and age. Thus, we conducted an online survey for this study. The exclusion criteria were having a diagnosis of an intellectual disability, dementia, or positive symptoms of schizophrenia; being at imminent risk of suicide; or being otherwise determined unfit for participation by a therapist or researcher. A web-based survey company was hired to conduct the survey, and patients with various mental disorders registered to complete it. Participants were recruited through the survey company’s website. Based on the information provided by the registrants, the survey company selected those who met the inclusion criteria and sent them an email requesting their cooperation in the study. The title of the survey was “Questionnaire on the way of thinking and feeling about mental disorders.” The study was explained in writing, and submitting a completed questionnaire was deemed to constitute consent. Two weeks after submitting the survey, some participants completed the questionnaire again, reviewed the explanation of the study, and provided additional formal consent. Shimizu (2018) found that the minimum sample size in an exploratory factor analysis is more than 100, and that in many studies, it is important to conduct random sampling. In the present study, we recruited participants online, so that the number of participants could be more than 100 within a certain period. In order to determine the sample size for the confirmatory factor analysis, an *a priori* power analysis was conducted. For the power analysis, the findRMSEAsamplesize function (Jorgensen et al., 2018) of semTools was used in R (browser version). The findRMSEAsamplesize function is a function that performs the power analysis of the covariance structure analysis. Since confirmatory factor analysis was conducted by covariance structure analysis in this study, the power analysis using the findRMSEAsamplesize function was considered appropriate. The degrees of freedom for the conformity factor analysis was 151. The power of the test was set at 0.80, and the alpha error at 0.05. The parameter of the final findRMSEAsamplesize function was set to (rmsea0 = 0, rmseaA = 0.05, df = 151, a 0.80, 0.05). As a result of the analysis, the calculated sample size was 130.

### Materials

#### Demographic Data

The participants were asked to provide personal data, including their sex, age, occupation (full-time, part time, unemployed, and student), and education (college or above, high school, junior high school). They were also asked to provide information on their marital status, parenting status (whether they have children), diagnosis, history of hospitalization for psychiatric treatment, history of involuntary hospitalization, and the period since first receiving treatment for a mental health issue.

The following scales were used in this study: (1) the Japanese version of the BACE v3, (2) the Stigma Scale for Receiving Psychological Help (SSRPH), and (3) the Japanese version of the Internalized Stigma of Mental Illness (ISMI). We used the same procedure to translate the BACE v3 and SSRPH into Japanese. After receiving permission from the authors of each scale, the English versions of the BACE v3 and the SSRPH were translated to Japanese by two authors who speak Japanese as their first language and English as their second language; one author is a clinical psychologist with a Ph.D. who has experience translating an English book on schema therapy to Japanese, while the other author is a bilingual graduate student majoring in clinical psychology.

#### Japanese Version of the Barriers to Access to Care Evaluation Scale Version 3

The BACE v3 is a measurement instrument composed of 30 items which are scored ranging from 0 (not at all) to 3 (a lot). Higher scores represent a greater barrier to seeking treatment. The treatment stigma subscale consists of 12 items, and the mean of the 12 items is used as the treatment stigma subscale score. Accordingly, Clement et al. (2012), Cronbach’s alpha for the treatment stigma subscale of the BACE v2 was 0.89, which indicates good internal consistency. Furthermore, Lin’s concordance statistic was ρc = 0.816, which surpasses the criterion of 0.70 for acceptable test-retest reliability. The subscale of BACE v2 consisted of 13 items; however, as one item was similar to others, it was removed when creating the BACE v3. Therefore, there is no loss of reliability for the stigma subscale between the BACE v2 and BACE v3.

To ensure cross-cultural validity when creating the Japanese version of the BACE v3, the procedure followed the Manual for Researchers made by the original author to explain how to use and translate the BACE v3 to different languages. After being translated to Japanese, the scales were back translated into English by two psychologists from a translation agency whose first language is English and second language is Japanese. One of the translators translated the Japanese version to English without looking at the original scale, and another translator compared the original scale with the translated English version, noting any items where differences occurred.

Our study team met to discuss the back-translated version and compared it with the original English version, leading to a list of disputed items needing further discussion. First, we discussed the use of the word “ethnic” in item 15. In Japanese society, where the population of foreign citizens is only 2%, the concept of “ethnic” is unfamiliar, but the concept of “cultural,” which refers to locality—regardless of ethnicity—is more well-known. Thus, the word “ethnic” was excluded and “cultural” was retained in the Japanese translation. Item 19, which was also disputed, had the phrase “take me seriously,” which could not be translated into Japanese verbatim. Thus, a different phrase was used, but the item was adjusted to have the same nuance. The back-translation of the Japanese version yielded the phrase, “be treated as a normal person by others.” The completed back-translations were presented to the original authors, and permission was obtained to use them only for this study.

In the next step, to confirm face validity, a pilot survey was conducted. Six people (one male and five females, age range 36–53, average age = 42.22) checked and answered the draft of the Japanese version of the BACE v3. Their self-reported primary diagnoses were depression (2), anxiety (1), and neurodevelopmental disorder (3). Two of the authors asked them to list queries about the scales and to note any difficulties they experienced when completing the draft scales. As no questions or suggestions were provided by the participants, the face validity of the Japanese version of the BACE v3 was ascertained.

#### Stigma Scale for Receiving Psychological Help

The SSRPH is a measure of the stigma associated with receiving psychological treatment, comprising five items (Komiya et al., 2000). Each item is scored from 0 (strongly disagree) to 3 (strongly agree), with a higher score indicating greater treatment stigma. Its internal consistency is alpha = 0.72. The SSRPH was used to confirm convergent validity. Based on previous research (Clement et al., 2012), a moderate positive correlation between BACE v3 and SSRPH was expected to be found. Upon receiving approval from the author of the SSRPH to translate the scale to Japanese, it was translated in the same way as the BACE v3. As the author of the SSRPH is Japanese, we asked him to confirm the Japanese version after our translation and obtained his approval.

#### Japanese Version of the Internalized Stigma of Mental Illness

The ISMI (Ritsher et al., 2003) is a measure of the internalized stigma of people with a mental illness and consists of 29 items. Each item is scored from 1 (strongly disagree) to 4 (strongly agree), and a high score indicates high internalized stigma. Using all 29 items from the ISMI Japanese version (Tanabe et al., 2016), the same scoring system was applied. The Japanese version of the ISMI has strong internal consistency (α = 0.91) and test-retest reliability (*r* = 0.85). The Japanese version of the ISMI was used to confirm convergent validity. Based on previous research (Clement et al., 2012), a moderate positive correlation between the BACE v3 and the Japanese version of the ISMI was expected to be found.

### Ethics

We provided the aim of the study and an informed consent form on the cover of the questionnaire on the website. The participants were also asked whether they agreed to participate in the study. These procedures were approved by the Ethics Committee of Chiba University (No. 3199). All the methods were carried out in accordance to the guidelines of the university and the ethics committee.

### Procedure

#### Participants

From late November to mid-December 2018, 268 people answered the survey. All responses from the participants (130 men and 138 women, average age = 43.7 years, SD = 11.3 years) were used for analysis. Of these, 220 participants (114 males and 106 females, average age = 44.3 years, SD = 11.2 years) responded to the Japanese version of the BACE v3 2 weeks after first completing the survey. The demographic characteristics of the participants are shown in Table 1.

**TABLE 1:** Participant sociodemographic and clinical characteristics.

Variable		*N*	%
Gender (*n* = 268)	Male	130	48.5
	Female	138	51.5
Age (*n* = 268)	Mean (SD) = 43.7 (11.3)	Range =	20–65
Highest level of education (*n* = 268)	Higher education	122	45.5
	High school	101	37.7
	Junior high school	19	7.1
	Other	26	9.7
Employment status (*n* = 268)	Work full-time	72	26.9
	Work part-time	51	19.0
	Housewife/househusband	41	15.3
	Student	3	1.1
	Not working	92	34.3
	Other	9	3.4
Relationship status (*n* = 268)	Single	153	57.1
	Married	83	31.0
	Divorced, separated, or widowed	32	11.9
Any children (including adult and non-resident children) (*n* = 268)	Yes No	77 191	28.7 71.3
Self-reported diagnosis (if more than one, first listed) (*n* = 268)	Schizophrenia/schizoaffective disorder	60	22.4
	Bipolar disorder	37	13.8
	Depression	42	15.7
	Anxiety disorder	81	30.2
	Personality disorder	6	2.2
	Neurodevelopmental disorder	45	16.8
Ever admitted to hospital for psychiatric treatment (*n* = 268)	Yes No	83 185	31.0 69.0
Years since first treatment for mental health problem	Mean (SD) = 12.2 (8.4)	Range =	1–44

Out of all the participants, 45.9% were employed on a full- or part-time basis. The most common self-reported primary diagnoses were schizophrenia and schizoaffective disorder (22.4%), followed by bipolar disorder (13.8%), depressive disorders (15.7%), anxiety disorders (30.2%), personality disorders (2.2%), and neurodevelopmental disorders (16.8%). In total, 31% of the participants had been previously hospitalized for mental health problems.

#### Statistical Analysis

As the online survey was set up to eliminate missing data, there were no missing data. The overall sample was divided into two parts; exploratory factor analysis (EFA) was performed on the first part to identify the initial factor structure (*N* = 134), and confirmatory factor analysis (CFA) was performed on the second part (*N* = 134). The data sample for the EFA was 67 males and 67 females, average age = 44.2 years, SD = 11.8 year. The data sample for the CFA was 63 males and 71 females, average age = 43.3 years, SD = 10.8 year. An EFA was performed using the maximum likelihood method with promax rotation, followed by a CFA to confirm the factor structure. We adopted the criteria recommended by Vandenberg and Lance (2000), as follows: Tucker–Lewis index (TLI) ≦ 0.90; standardized root mean square residual (SRMR) ≦ 0.15; and comparative fit index (CFI) ≧ 0.90. We used the following criteria for root mean square error of approximation (RMSEA): an RMSEA value less than or equal to 0.06 was considered a good fit;0.08 or less indicated reasonable fit; 0.08 to 0.10 indicated a mediocre fit; and values above 0.10 indicated a poor fit (Hooper et al., 2008; Schreiber, 2008). We used Cronbach’s alpha to examine the internal consistency of each of the subscales, and the intraclass correlation coefficient (ICC) to confirm test-retest reliability at 2-week intervals. We assessed the convergent validity of the treatment stigma subscale by calculating the Pearson product-moment correlation coefficient with the SSRPH and ISMI. Cohen’s (1988) criteria were used to determine the degree of correlation, considering | r| ≧ 0.10 to be a weak correlation. All data were analyzed using SPSS (Ver. 27.0) and AMOS (Ver. 25.0).

## Results

### Factor Analysis

Six items had checkboxes for “not applicable,” depending on whether a participant had children or a job; 45.9% of the sample was employed either full-time or part-time, and only 28.7% of those had children. Furthermore, only 13.1% of the total number of participants had both a job and children. This shows that the data count was inadequate due to the small number of respondents for the “work” and “child” condition items in the sample of this study. Therefore, an EFA was conducted to confirm the factor structure of the Japanese version of the BACE v3 by excluding the six items referring to employment and children. The excluded items were item 5 (“Concern that it might harm my chances when applying for jobs”), item 14 (“Concern that I might be seen as a bad parent”), item 24 (“Concern that my children may be taken into care or that I may lose access or custody without my agreement”), item 27 (“Difficulty taking time off work”), item 28 (“Concern about what people at work might think, say, or do”), and item 29 (“Having problems with childcare while I receive professional care”).

To confirm the structure of the BACE v3, factor analysis was conducted on all 24 items. Maximum-likelihood method analyses using promax rotation were performed on the total exploratory sample (*N* = 134). Two factors were determined to be optimal from the scree plot. Except for five items, the factor loadings were above the general criterion of 0.350. The factor loadings of five items were less than 0.350, which was considered sufficiently low. Thus, these five items were excluded: item 4 (“Fear of being put in hospital against my will”), item 23 (“Preferring to get help from family or friends”), item 25 (“Thinking the problem would get better by itself”), item 11 (“Not being able to afford the financial costs involved”), and item 7 (“Thinking I did not have a problem”). A factor analysis was conducted again with the remaining 19 items. Finally, all eight items loading on the first factor were original treatment stigma items, and all eleven items loading on the second factor were original non-stigma items. Following the original version, the first factor was the treatment stigma subscale and the second was the non-stigma subscale. The factor loadings, mean scores, and SDs of each item for the two-factor model are shown in Table 2.

**TABLE 2:** Mean scores, SD, and factor loading for each item in the Japanese version of the BACE v3.

Item		Stigma	Non-stigma	Mean	SD
3	Concern that I might be seen as weak for having a mental health problem	**0.589**	0.133	1.48	0.97
8	Concern about what my family might think, say, do, or feel	**0.410**	0.275	1.22	0.91
9	Feeling embarrassed or ashamed	**0.610**	0.275	1.26	0.88
12	Concern that I might be seen as “crazy”	**0.780**	−0.06	1.40	0.97
17	Concern that people I know might find out	**0.860**	−0.117	1.13	0.93
19	Concern that people might not take me seriously if they find out I was receiving professional care	**0.947**	−0.158	1.34	0.99
21	Not wanting a mental health problem to be on my medical records	**0.710**	−0.058	1.33	1.00
26	Concern about what my friends might think, say, or do	**0.720**	−0.107	1.28	0.97
1	Being unsure where to go to get professional care	0.060	**0.600**	1.19	0.94
2	Wanting to solve the problem on my own	−0.044	**0.580**	1.59	0.95
6	Problems with transport or traveling to appointments	−0.193	**0.451**	0.97	0.94
10	Preferring to get alternative forms of care (e.g., traditional/religious healing or alternative/complementary therapies)	−0.094	**0.483**	0.65	0.78
13	Thinking that professional care probably would not help	−0.025	**0.576**	0.96	0.82
15	Professionals from my own cultural group not being available	−0.034	**0.726**	0.72	0.77
16	Being too unwell to ask for help	0.041	**0.594**	0.99	0.82
18	Dislike of talking about my feelings, emotions, or thoughts	0.287	**0.448**	1.19	0.97
20	Concerns about the treatments available (e.g., side effects of medication)	0.233	**0.428**	1.40	0.94
22	Having had previous bad experiences with professional care for mental health	0.022	**0.499**	1.29	1.04
30	Having no one who could help me get professional care	0.024	**0.749**	1.07	0.91

*The number in the far-left column indicates the item number. The loadings of 0.350 or above are boldfaced.*

**FIGURE 1:**
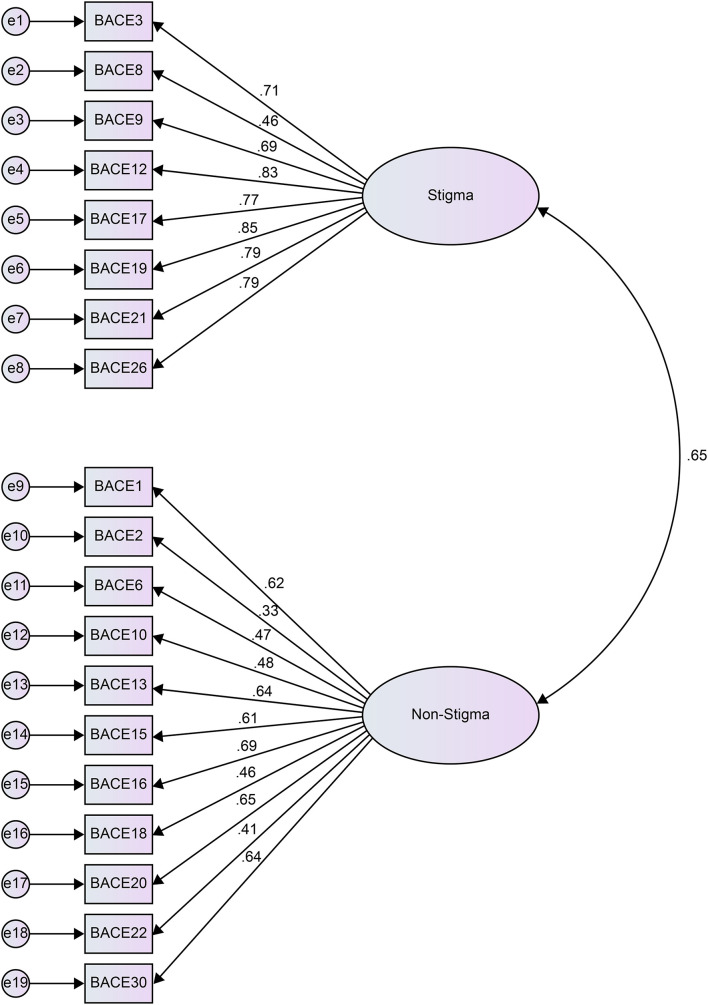
Result of the confirmatory factor analysis of the Japanese version of the BACE v3.

To test the suitability of the structure proposed by the EFA, we conducted a CFA. Confirmatory factor analysis was performed on the confirmatory sample (*N* = 134). This was performed using a hypothetical model with the same two factors as the original version and items corresponded to each factor. The results showed the following fit indices: χ^2^ (151) = 303.14 (*p* < 0.01); RMSEA = 0.087; TLI = 0.842; SRMR = 0.078; and CFI = 0.86. Based on the relevance criteria adopted by Vandenberg and Lance (2000), the values obtained were not good enough, but were not bad either. As shown in Figure 1. It can be concluded that the same two factors are valid, as in the original version. There are eight items that make up the BACE treatment stigma subscale, which is consistent with the treatment stigma subscale items in the original version of the BACE v3. However, as four conditional items in the treatment stigma subscale of the original version of the BACE v3 were removed in advance, these are not included in the current treatment stigma subscale. In the original version of the BACE v3, the scoring for the treatment stigma subscale is the rating of the mean of the response items on the stigma subscale. In the Japanese version of the BACE, the scoring for the treatment stigma subscale is the total score of stigma-related barrier ratings, as all participants responded to the same items given the exclusion of conditional items in the study.

### Internal Consistency and Reliability

Cronbach’s alpha for the treatment stigma subscale was α = 0.90, indicating good internal consistency. Cronbach’s alpha for the non-treatment stigma subscale was α = 0.83, indicating good internal consistency.

The ICC was calculated to examine the test-retest reliability. The results showed that the ICC value for the treatment stigma subscale was 0.76 [confidence interval (CI): 0.70–0.81]. These values are generally considered to be favorable (Landis and Koch, 1977). The results showed that the ICC value for the non-treatment stigma subscale was 0.64 (CI: 0.56–0.71). These values are generally considered to be substantial (Landis and Koch, 1977).

### Validity

For convergent validity, there was a strong positive correlation between the total score of the BACE treatment stigma subscale and the total score of the SSRPH (*r* = 0.66, *p* < 0.01) and the total score of the ISMI (*r* = 0.58, *p* < 0.01). The results showed that the subscales are convergent, and, therefore, have construct validity.

## Discussion

In the present study, we investigated the factor structure, internal consistency, reliability, and validity of the treatment stigma subscales of the Japanese version of the BACE v3. Two factors were extracted: stigma-related barriers and non-stigma-related barriers. The results were consistent with those of the original paper, which conceptually created the factors. The results of this CFA did not show the best agreement between the model and the data. While the BACE v3 assesses barriers to treatment for people with mental health issues, it is designed primarily to measure treatment stigma, which forms one of its subscales. The other factor derived from the EFA, the non-stigma subscale, consists of items unrelated to stigma that are considered barriers to treatment, such as treatment concerns, availability, accessibility, acceptability, and affordability; therefore, it is not a theoretically unified factor and the entire BACE v3 scale does not have a theoretical basis. While examining the treatment stigma subscale, both the internal consistency and test-retest reliability of the treatment stigma subscales showed favorable values, indicating adequate reliability. There was also a significant positive correlation between the total score of the Japanese version of the treatment stigma subscale of the BACE v3 and SSRPH and the total score of the Japanese version of the treatment stigma subscale of the BACE v3 and ISMI, confirming the convergent validity of the BACE v3. The strength of this scale is that the Japanese version of BACE v3 will be able to measure barriers to professional mental healthcare for patients with all types of mental illnesses in Japan. In particular, the quantitative measurement of treatment stigma enables screening for stigma and provides treatment strategies that are focused on stigma.

In this study, it was unclear whether the six conditional items, which depended on whether the participant had children and/or employment, were loaded on any of the factors because they were not included in the factor analysis due to the insufficient sample size. Four of these six items—items 5, 14, 24, and 28—were classified as stigma-related barriers, while two items—items 27 and 29—were classified as non-stigma-related barriers. Next, we conducted an EFA in which we removed five items (items 4, 7, 11, 23, and 25) whose loadings did not meet the 0.350 criteria for any of the factors. These five items were classified as non-stigma-related barrier factors in the original study (Clement et al., 2012). Item 4 can be interpreted as a concern about professional mental health institutions. For items 4, 7, 11, 23 and 25, the low loadings for either factor may have been because the participants in this study had received treatment from a mental health professional and were already aware of their problems.

To test the convergent validity of the treatment stigma subscale of the Japanese version of the BACE v3, we used the SSRPH, a stigma scale for receiving psychological assistance, and the ISMI, a measure of the internalized stigma of mental illness. These measures are the same as those used for the BACE v3 in the original article (Clement et al., 2012). In the present study, the correlations between the treatment stigma subscale and the SSRPH and ISMI were 0.66 and 0.58, respectively, while the correlations were 0.30 and 0.40, respectively, in the original study (Clement et al., 2012). The SSRPH and ISMI do not have grading items for “evaluation as a parent” or “evaluation by peers.” In this study, the removal of four conditional items related to children and work from the analysis, which were among the items that constituted the treatment stigma subscale in the original study (Clement et al., 2012), may have been one of the factors that resulted in a strong correlation compared to the original study.

There are four limitations to this study. First, this study utilized a web-based survey, which may have resulted in bias regarding participant attributes. Therefore, in the future, we believe that it is necessary to reduce any bias in the participants’ attributes by conducting surveys through means other than web-based surveys, such as interviews, detention surveys, mail surveys, and group surveys. Second, in this study, the analysis excluded six conditional items related to children and employment due to the small sample size. In the future, a survey should be conducted wherein the participants are able to provide responses regarding children and employment so that an analysis that includes all items may be performed. Third, five items were removed from the Japanese version of the BACE v3 on the grounds that their factor loadings were below 0.350. It is possible that the participants in this study were influenced by the fact that they had received treatment from a mental health professional and were already aware of their problems. To continue refining the Japanese version of the BACE v3 after including these five items, a survey of people who need professional mental healthcare but have not yet received it must be conducted. Finally, although the psychometric properties of the SSRPH, have been confirmed in previous studies (Komiya et al., 2000), no studies have confirmed its reliability and validity in a Japanese population. The validation of the SSRPH in a Japanese population will strengthen the evidence regarding the validity of the Japanese version of BACE v3.

## Conclusion

The BACE v3 has acceptable consistency, reliability, and validity for the assessment of barriers to access to mental health care, including treatment stigma among people with mental disorders in Japan. It provides a comprehensive scale for quantitatively measuring treatment stigma as well as behavioral barriers, and for using specialized treatment facilities for mental health.

## Data Availability Statement

The raw data supporting the conclusions of this article will be made available by the authors, without undue reservation.

## Ethics Statement

The studies involving human participants were reviewed and approved by Ethics Committee of Chiba University (No. 3199). The patients/participants provided their written informed consent to participate in this study.

## Author Contributions

MH and FO collected the data. HN and MH conducted statistical analyses and interpretation of data. MH, FO, and MS critically revised the manuscript. FO, TO, and ES supervised the study. All authors read and approved the final manuscript.

## Conflict of Interest

The authors declare that the research was conducted in the absence of any commercial or financial relationships that could be construed as a potential conflict of interest.

## Publisher’s Note

All claims expressed in this article are solely those of the authors and do not necessarily represent those of their affiliated organizations, or those of the publisher, the editors and the reviewers. Any product that may be evaluated in this article, or claim that may be made by its manufacturer, is not guaranteed or endorsed by the publisher.

## Abbreviations

CFA: confirmatory factor analysis
CI: confidence interval
EFA: exploratory factor analysis
ICC: intraclass correlation coefficient
ISMI: Internalized Stigma of Mental Illness
SSRPH: Stigma Scale for Receiving Psychological Help
RMSEA: root mean square error of approximation
TLI: Tucker–Lewis index.
